# Physico-chemical and high frequency monitoring dataset from mesocosm experiments simulating extreme climate events in lakes

**DOI:** 10.1016/j.dib.2023.109302

**Published:** 2023-06-07

**Authors:** Viet Tran-Khac, Philippe Quetin, Laurent Espinat, Laura Crépin, Charlotte Cousin, Pascal Perney, Jean-Christophe Hustache, Geneviève Chiapusio, Isabelle Domaizon, Serena Rasconi

**Affiliations:** Université Savoie Mont Blanc, INRAE, CARRTEL, 75 bis, avenue de Corzent, Thonon les Bains, France

**Keywords:** Climate change, Aquatic ecosystems functioning, Turbid storms, Terrestrial subsidies, Experimental ecology, Large peri-alpine lakes, Environmental parameters

## Abstract

We present two datasets composed of high frequency sensors data, vertical *in situ* profiles and laboratory chemical analysis data, acquired during two different aquatic mesocosm experiments performed at the OLA (“Long-term observation and experimentation for lake ecosystems”) facility at the UMR CARRTEL in Thonon les Bains, on the French shore of Lake Geneva. The DOMLAC experiment lasted 3 weeks (4-21 October 2021) and aimed to simulate predicted climate scenarios (i.e. extreme events such as storms and floods) by reproducing changes in quality and composition of lake subsidies and runoff by increased inputs of terrestrial organic matter. The PARLAC experiment lasted 3 weeks (5-23 September 2022) and aimed to simulate turbid storms by light reduction.

The experimental setup consisted of nine inland polyester laminated tanks (2.1 m length, 2.1 m width and 1.1 m depth) with a total volume of approximately 4000 L and filled with water directly supplied from the lake at 4m depth. Both experimental design included three treatments each replicated three times. The DOMLAC experiment involved a control treatment (no treatment applied) and two treatments simulating allochthonous inputs from two different dissolved organic matter (DOM) extract from peat moss *Sphagnum sp*. (Peat-Moss treatment) and *Phragmites australis* (Phragmite treatment). The PARLAC experiment involved a control treatment (no treatment applied) and two treatments simulating two different intensity of light reduction. In the Medium treatment transmitted light was reduced to 70% and in the High treatment transmitted light was reduced to 15%.

The datasets are composed of: 1. *In situ* measures from automated data loggers of temperature, conductivity, dissolved oxygen and CO_2_ acquired every 5 minutes at 0.1, 0.5 and 1 m depth (DOMLAC) and 0.5m (PARLAC) for the entire period of the experiment. 2. *In situ* profiles (0-1 m) of temperature, conductivity, pH, dissolved oxygen (concentration and saturation) acquired twice a week during the experiment. 3. *In situ* measures of light spectral UV/VIS/IR irradiance (300-950 nm wavelength range) taken in the air and at 0, 0.5 and 1 m twice a week on the same day of the profiles at point 2. 4. Laboratory chemical analysis of integrated samples taken twice a week on the same day of the *in situ* profiles at point 2 and 3 of conductivity, pH, total alkalinity, NO_3_, total and particulate nitrogen (Ntot, Npart), PO_4_, total and particulate phosphorus (Ptot, Ppart), total and particulate organic carbon (TOC, POC), Ca, K, Mg, Na, Cl, SO_4_ and SiO_2_. Only for DOMLAC also analyses of NH_4_, NO_2_ and dissolved organic carbon (DOC). 5. Laboratory analysis of pigments (Chl*a*, Chl*c*, carotenoids, phaeopigments) extracted from samples collected at point 4. 6. Only for DOMLAC, specific absorbance on the range 600-200nm of DOM (i.e. <0.7 µm) measured on samples collected at point 4.

This dataset aims to contribute our understanding of how extreme climate events can alter lake subsidies and affect the regulation of ecosystem processes such as production, respiration, nutrient uptake and pigment composition. The data can be used for a wide range of applications as being included in meta-analysis aiming at generalising the effect of climate change on large lakes including simulating future scenarios in a broad range of geographical areas as we used different inputs of DOM leached from litters reproducing catchments characteristics typical of different latitudes, such as mostly dominated by large leaf forests and phragmites at middle latitude, and coniferous forests rich of peat mosses that spread along the water surface typical of Northern regions.

**Specifications Table**

**Table:** 

Subject	Environmental Science - Ecology
Specific subject area	Physico-chemical and high frequency monitoring datasets produced during two grounded base mesocosm experiments directly supplied with Lake Geneva water
Type of data	Tables
How data were acquired	**Continuous:** Temperature (Therm107), conductivity (CS547A-L), CO_2_ (GMP252 - Vasaila), PAR (Li-93): Campbell Scientific Temperature: HOBO Temperature, dissolved oxygen (concentration and saturation): Minidot **Probes:** Temperature, conductivity, turbidity, dissolved oxygen (concentration and saturation): CTD 90M – (Sea & Sun Technology GmbH) Light irradiance (300-950 nm): RAMSES-ASC-VIS **Laboratory analysis according**[1]: Conductivity and pH: ISO 10523:2008 Total alkalinity: ISO 9963-1 NH_4_^+^, NO_2_^-^ , Ntot and Npart: ISO 5664:1984; ISO 6777:1984; ISO 10304- 1; EN 12260:2003; ASTM D2579-93e1 PO_4_^3-^, Ptot and Ppart: EPA 365.3 POC, DOC and TOC: ASTM D2579-93e1 Ca^2+^, K^+^, Mg^2+^ and Na^+^: NF 90-020 NO_3_^-^, Cl^-^ and SO_4_^2-^: ISO 10304-1 SiO_2_: NF T90-007 Pigments and Specific Absorbance: ISO 10260: 1992
Data format	Raw
Description of data collection	Data from automated data loggers were acquired every 5 minutes at 0.1, 0.5 and 1 m depth during the 3 weeks of the DOMLAC experiment (October 4–22, 2021) and at 0.5 m depth during the 3 weeks of the PARLAC experiment (September 5 – 23, 2022). *In situ* profiles and samples for lab analysis were acquired twice a week on the same day in every mesocosm from the surface (0 m) to 1 m (maximum depth of the mesocosms). Integrated samples (0-1 m) were taken using a syphon.
Data source location	Institution: UMR INRAE CARRTEL City/Town/Region: Thonon les Bains Country: France GPS coordinates: 46.368, 6.454
Data accessibility	The dataset described in this data paper is accessible as open file in the Data INRAE repository https://entrepot.recherche.data.gouv.fr/dataverse/inrae DOMLAC dataset uploaded in the repository as single excel file [2]: Repository name: Data INRAE Direct URL to data: https://doi.org/10.57745/QJEJIJ PALAC dataset uploaded in the repository as single excel file [3]. Repository name: Data INRAE Direct URL to data: https://doi.org/10.57745/N2NKA2
Related research article	Complementary DIB articles on a different *in situ* mesocosm experiment performed in the same lab and collecting data using the same methods: [4] Tran-Khac V, Quetin P, Domaizon I, Jacquet S, Espinat L, Gallot C, Rasconi S. 2020. *In situ* pelagic dataset from continuous monitoring: a mesocosm experiment in Lake Geneva (MESOLAC). Data in Brief. 32:106255. https://doi.org/10.1016/j.dib.2020.106255 The complementary dataset published in [4] produced during the same experiment is available at: https://data.inra.fr/dataset.xhtml?persistentId=doi:10.15454/T3VCB0 [5] Tran-Khac V, Perney P, Crépin L, Quetin P, Domaizon I, Jacquet S, Espinat L, Gallot C, Rasconi S. 2021. Physico-chemical dataset from an *in situ* mesocosm experiment simulating extreme climate events in Lake Geneva (MESOLAC). Data in Brief. 36:107150. https://doi.org/10.1016/j.dib.2021.107150 The complementary dataset published in [5] produced during the same experiment is available at: https://data.inrae.fr/dataset.xhtml?persistentId=doi:10.15454/PCOPYW

## Value of the Data

• These datasets improve our understanding of the effect of environmental forcing on lake physico-chemical characteristics (such as temperature, oxygen and nutrient concentration) under simulated intense weather events. Additionally, the DOMLAC study evaluated the impact of increased input of Dissolved Organic Matter leached from different litter types such as peat moss and phragmites, representative of different geographic areas and reproducing organic matter gradients from Northern to middle latitudes.
• This open and raw dataset will benefit the scientific community working on environmental ecology as can be used for a wide variety of applications including further meta-analysis aiming at generalising the effect of climate change across different regions and latitudes including catchments and lake shores with different characteristics.
• These data provide a quality-assured baseline of key lake parameters measured using standardised methods that may help to discern changes in lake environmental status and assist in better managing and protecting lake ecosystems.

## 1 Objective

In the context of global warming, extreme precipitation and pluvial flooding are predicted to increase [6]. Persistent storm-induced changes in water clarity could be at least as important as rising air temperatures in determining lake responses to climate change [7,8].

We initiated our mesocosm research during summer 2019 by running an *in situ* experiment in Lake Geneva [4,5] to simulate extreme events such a turbid storms. In the experimental treatments we applied manual mixing, increased DOC concentration and reduced light on two different intensity of stress (intermediate and intense) to test the effect on plankton diversity and the related functional traits. The intense treatment strongly affected the oxygen concentration [4] and community pigment composition [5]. The exchange of organic matter among ecosystems showed major impacts on plankton populations structure and dynamics [9], yet little is known about how individual responses combine with the ecosystem feedback. To further investigate these questions we setup a ground based mesocosm system directly supplied with lake water and specifically designed to provide a comprehensive panel of measures that can be generalized to different temporal and spatial scales.

The projects DOMLAC and PARLAC were designed in the continuity of a series of different and complementary mesocosm experiments aiming to simulate future scenarios and disentangle how predicted increase precipitation affects plankton community composition, trophic transfer and the related ecosystem feedback on functional and metabolic process such as production, respiration and recycling. During extreme weather events different forcing are often occurring at the same time. The specific effect of stressors such as organic matter pulses and reduction in light penetration can be thus masked if one stressor acts in a stronger way. We simulated future scenarios by applying separately the main stressors occurring during turbid storms, such as different OM quality inputs (DOMLAC project) and light limitation (PARLAC) to understand the specific effect of each stressor.

Combining high-frequency monitoring with discrete measures, will allow elucidating the effect of extreme events at different scales (temporal, spatial) and organizational levels (community and food web).

## 2 Data Description

The datasets described in this section can be download at data repository INRAE. The data are stored as single excel file containing six (DOMLAC) and five (PARLAC) data sheets and two summary table sheets [2,3].

In the DOMLAC dataset [2] the first rows of the sheets contain indication on the used device (row flagged by “# Device”), the depth of the measure (row flagged by “# Depth”) and the unit of the measure (row flagged by “#”). Unique ID for each column (Sample_Name) includes the mesocosm treatment (named Control, Phragmite and Peat-Moss), replicate (from 1 to 3) and the sampling event (numbered S1 for 04.10.2021, S2 for 07.10.2021, S3 for 11.10.2021, S4 for 14.10.2021, S5 for 18.10.2021 and S6 for 21.10.2021), i.e. first row named S1_Control1 is the Control treatment, replicate 1 on the sampling day 1 04.10.2021. Samples labelled with “S1+” in the column “Sample_Name” and “Sampling” are the TOC measures taken at the surface of all mesocosms few minutes after the DOC solution was added on 6^th^ of October 2021. Complete names abbreviations and sampling dates are in Table 1.

**Table 1:** DOMLAC dataset [2] mesocosms names abbreviations and sampling dates.

Treatment short	Treatment	Sampling	Date
C1	Control1	S1	04/10/21
C2	Control2	S1+	06/10/21
C3	Control3	S2	07/10/21
P1	Phragmite1	S3	11/10/21
P2	Phragmite2	S4	14/10/21
P3	Phragmite3	S5	18/10/21
PM1	Peat-Moss1	S6	21/10/21
PM2	Peat-Moss2		
PM3	Peat-Moss3		

Missing data and values below the limit of quantification are respectively identified by the code NA and LOQ.

In the PARLAC dataset [3] the first rows of the sheets contain indication on the used device (row flagged by “# Device”), the depth of the measure (row flagged by “# Depth”) and the unit of the measure (row flagged by “#”). Unique ID for each column (Sample_Name) includes the mesocosm treatment, named Control, Medium (Medium light reduction, i.e. 70% transmitted light) and High (high light reduction, i.e. 15% transmitted light), replicate (from 1 to 3) and the sampling event (numbered S1 for 05.09.2022, S2 for 08.09.2022, S3 for 12.09.2022, S4 for 15.09.2022, S5 for 19.09.2022 and S6 for 22.09.2022), i.e. first row named S1_Control1 is the Control treatment, replicate 1 on the sampling day 1 05.09.2022. Complete names abbreviations and sampling dates are in Table 2.

**Table 2:** PARLAC dataset [3] mesocosms names abbreviations and sampling dates.

Treatment short	Treatment	Sampling	Date
C1	Control1	S1	05/09/2022
C2	Control2	S2	08/09/2022
C3	Control3	S3	12/09/2022
M1	Medium1 (70% transmitted light)	S4	15/09/2022
M2	Medium2 (70% transmitted light)	S5	19/09/2022
M3	Medium3 (70% transmitted light)	S6	22/09/2022
H1	High1 (15% transmitted light)		
H2	High2 (15% transmitted light)		
H3	High3 (15% transmitted light)		

Missing data and values below the limit of quantification are respectively identified by the code NA and LOQ.

Both dataset [2,3] are constituted of the following parts:

**Continuous monitoring data:** The first sheet (named “Continuous”) contains data acquired continuously every 5 minutes from automated data loggers placed at the depths of 0.1 m (Surface - S), 0.5 m (Middle - M) and 1 m (Bottom - B) in each mesocosm.

The list of data loggers with respective measured parameters and units is provided below:

- Campbell Scientific: Temperature (Therm107) - °C, Conductivity CS547A-L - µScm^-1^, pCO_2_ GMP252 – ppm, PAR (Licor LI-193) - μmols^-1^m^-2^
- HOBO: Temperature (HOBO onset) - °C
- Minidot: Temperature - °C, dissolved oxygen (concentration and saturation) – mgO_2_L^-1^, %

***In-situ* vertical profiles data:** The second sheet (named “CTD”) contains *in situ* profiles (0-1 m) acquired in every mesocosm twice a week for the entire duration of the experiment using the multiparameter probe CTD 90M – (Sea & Sun Technology GmbH).

Measured parameters and measure unit include:

Conductivity – µScm^-1^
Temperature – °C
Turbidity – NTU (Nephelometric Turbidity Units)
Photosyntethic Active Radiation (PAR) – μmol (m^−2^s^−1^)
Oxygen saturation – %
Oxygen concentration – mgO_2_L^-1^

**Light data:** The third sheet (named “Light”) contains *in situ* measures of light spectral UV/PAR/IR irradiance (300-950 nm wavelength range) acquired using RAMSES-ASC-VIS irradiance sensor at 0, 0.5 and 1 m on the same day of the CTD *in situ* vertical profiles. Measures are taken as mW m^−2^nm and converted by manufacturer (triOS GmbH) spectral calibration to physical units (power scale/quanta scale), i.e. expressed as μmol (m^−2^s^−1^nm^−1^).

**Integrated samples data:** The fourth sheet (named “Chemical”) contains data from laboratory chemical analysis according [1] on integrated samples (0-1 m) collected on the same day of *in situ* vertical profiles.

Measured parameters and measure unit, instruments and protocols include:

Conductivity (Cond, µScm^-1^) measured using conductometric method with temperature corrections systematically applied when performing lab measurements of the conductivity (ISO 10523:2008).
pH measured with an automatic titrator (Basic Titrino 794, Metrohm ©) with a glass electrode.
Total alkalinity (TAC) measured by potentiometric determination (ISO 9963-1).

NH_4_^+^, NO_2_^-^, particulate and total nitrogen (Npart, Ntot, mgNL^-1^) measured using an ElementarVario© TOC/TNb coupled with a chemiluminescence detector (APNA-370; Horiba©) after high temperature digestion and catalytic post combustion (ISO 5664:1984; ISO 6777:1984; ISO 10304- 1; EN 12260:2003; ASTM D2579-93e1).

PO_4_^3-^ (mgPL^-1^), total and particulate phosphorus (Ptot and Ppart, mgPL^-1^) measured using A UV-vis spectrophotometer (Cary 50 scan; Varian©) following sulphuric acid digestion and a molybdenum blue method (EPA 365.3).

Total, dissolved organic carbon and particulate organic carbon (TOC, DOC, POC, mgCL^-1^) measured using a CHN elemental analyser following high-temperature combustion method with a gas chromatograph equipped with a thermal conductivity detector (Flash 2000; Thermoscientific©) (ASTM D2579-93e1).

Ca^2+^ (mgCa^2+^L^-1^), K^+^ (mgK^+^L^-1^), Mg^2+^ (mgMg^2+^L^-1^) and Na^+^ (mgNa^+^L^-1^) measured using flame (acetylene/air) atomic absorption spectrophotometry (AA240FS; Varian©). Lanthanum is added as a matrix modifier to minimize interference. (NF 90-020).

Cl^-^ (mgCl^-^L^-1^), NO_3_^-^ (mgNL^-1^), and SO_4_^2-^ (mg SO_4_^2-^L^-1^), quantified using ion exchange (861 Advanced Compact ion chromatograph; Metrohm©) with chemical suppression (ISO 10304-1).

SiO_2_ (mg SiO_2_L^-1^) analysed using a Smartchem 200 Discrete Analyzer (WESTCO Scientific Instruments©) (NF T90-007).

**Pigments data:** The fifth sheet (named “Pigments”) contains data from laboratory analysis of pigments extracted from integrated samples and analysed using spectrophotometry after extraction into 90% acetone (ISO 10260: 1992).

In the DOMLAC dataset [2] only:

**Specific Absorbance data:** The sixth sheet (named “Specific Absorbance”) contains data from specific absorbance measured in the range 200-600 nm from integrated samples filtered on 0.7 µm Glass fiber filters and analysed using spectrophotometry.

## 3 Experimental Design

The mesocosm experiments was performed at the OLA-CARRTEL ground based experimental facility, located in Thonon les Bains, France. Nine polyester laminated thanks of dimensions 2.1 m length, 2.1 m width and 1.1 m depth (total volume about 4000 L) are located on the shore of Lake Geneva and equipped with a pump and a pipe system which allows to fill simultaneously all experimental tanks with directly supplied lake water from 4m depth. The mesocosms were filled with a slow flow for about 8h and left to acclimate to the new conditions during one week (DOMLAC from 27.09.2021 until 03.10.2021 and PARLAC from 29.08.2022 until 04.09.2022). For the DOMLAC experiment on the second day of acclimation each mesocosm was inoculated with 3 plankton net tows (50 µm mesh, 0-20m) randomly taken simultaneously in the lake in front of the experimental facility. During the acclimation all the mesocosms were covered with a mosquito net and in the DOMLAC experiment for the entire experiment. The PARLAC mesocosm were each covered with a 2.1x2.1m lid with 1.2X1.2 light film according the treatment. Artificial mixing was induced during the entire acclimation and experiments with direct air bubbles from continuously releasing compressed air from a tube (6 mm diameter) placed at the bottom and in the centre of the mesocosms.

The DOMLAC experiment aimed to simulate predicted climate scenarios (i.e. extreme events such as turbid storms) by introducing inputs of dissolved organic matter (i.e. < 0.7 µm) leached from different litters, respectively peat moss and phragmites for the two treatments. The increase was done using the lake as reference (i.e. total DOC ∼ 1.2 mgL^−1^), and aiming at simulating the highest values measured in the Dranse tributary river during a flood, corresponding to 5x increased concentration, i.e. total DOC ∼ 6 mgL^−1^.

The DOM extract was prepared using commercial bio peat moss (*Sphagnum sp.*, Belgian company DCM) and phragmites (*Phragmites australis)* collected on the Lake Geneva surroundings. The plants were dried in the air for 48h and grinded before being processed. 250g of plants or peat bog were mixed with 1.5L of ultra pure water and autoclaved for one hour at 120 °C. This solution was then centrifuged for 15 min at 3500 r/min and the supernatant filtered through 0.7 μm glass fiber filters. The final solution was autoclaved again before being added to the mesocosms.

The experiment lasted 3 weeks (4-22 October 2021), the first sampling (S1) took place on 04.10.2022 after the environmental acclimation (no treatments were applied yet at that moment). Treatments were applied on the 06.10.2022 by adding as single pulse the peat moss extract to the Peat-Moss treatments and the phragmites extract to the Phragmite treatment.

The PARLAC experiment aimed to simulate turbid storms by manipulating transmitted light using LEE ND (Neutral Density) filters, which reduce light without changing colour. The experimental design included three treatments each replicated three times: a control treatment (covered with a ∼95% transmitted light filter to expose all the mesocosms to the same covering condition) and two different treatments simulating two different intensity of light reduction. In the Medium treatment transmitted light was reduced to ∼70% and in the High treatment transmitted light was reduced to ∼15%. The experiment lasted 3 weeks (5-23 September 2022), the first sampling (S1) took place on 05.09.2022 after the environmental acclimation (no treatments were applied yet at that moment). After the first sampling the treatments were applied by placing the light filters on all the mesocosms.

## 4 Instrumentation

Continuous monitoring data were acquired using data loggers including:

Temperature: Campbell, HOBO, MiniDOT
Conductivity: CS547A-L - Campbell Scientific
Dissolved oxygen: MiniDOT
CO_2_: Vaisaila GMP252

MiniDOT and HOBO are autonomous sensors and were ready for deployment. Temperature, conductivity and CO_2_ (CS547A-L - Campbell Scientific) are analog sensors and needed to be connected to data loggers. In the DOMLAC experiment data loggers Campbell CR10x were used in C1, CR1000 in Phragmite1 treatment and CR1000x for Peat-Moss1 treatment. In the PARLAC experiment data loggers Campbell CR10x were used in C1 and M1 treatment and CR1000 for H1 treatment.

Temperature sensors were calibrated in an environmental chamber. For Campbell and MiniDOT, calibration data were updated via the software.

Conductivity sensors were calibrated using a potassium chloride standard solution of 294 µS/cm at 25ºC. The calibration included temperature compensation.

Oxygen sensors were controlled at 100% saturation in water taking into account the barometric pressure.

CO_2_ sensors were calibrated in the air and in a closed chamber with standard gas at 500ppm. The intercalibration was done with certified reference CO_2_ sensor (AMT).

*In-situ* vertical profiles data for physical characterisation of each mesocosm included *in situ* profiles (0-1 m) of temperature, conductivity, pH, dissolved oxygen (concentration and saturation) acquired using a multiparameter probe CTD 90M. Light spectral measurements of UV, VIS, IR irradiance (300-950 nm wavelength range) were taken on the air above the mesocosm, at 0, 0.5 and 1 m using a RAMSES-ASC-VIS irradiance sensor.

Integrated samples (0-1 m) for laboratory chemical analysis were collected on the same day of *in situ* vertical profile data according protocols and methods detailed in [1].

Conductivity was measured using conductometric method. Temperature corrections are systematically applied when performing lab measurements of the conductivity (ISO 10523:2008). pH was measured with an automatic titrator (Basic Titrino 794, Metrohm ©) with a glass electrode.

Total alkalinity was measured by potentiometric determination (ISO 9963-1).

NH_4_^+^, NO_2_^-^ and total nitrogen were measured using an ElementarVario© TOC/TNb coupled with a chemiluminescence detector (APNA-370; Horiba©) after high temperature digestion and catalytic post combustion (ISO 5664:1984; ISO 6777:1984; ISO 10304- 1; EN 12260:2003; ASTM D2579-93e1).

PO_4_^3-^, total and particulate phosphorus were measured using a UV-vis spectrophotometer (Cary 50 scan; Varian©) following sulphuric acid digestion and a molybdenum blue method (EPA 365.3).

Dissolved and total carbon and particulate organic carbon measured using a CHN elemental analyser following high-temperature combustion method with a gas chromatograph equipped with a thermal conductivity detector (Flash 2000; Thermoscientific©) (ASTM D2579-93e1).

Ca^2+^, K^+^, Mg^2+^ and Na^+^ measured using flame (acetylene/air) atomic absorption spectrophotometry (AA240FS; Varian©). Lanthanum is added as a matrix modifier to minimize interference. (NF 90-020).

Cl^-^, NO_3_^-^ and SO_4_^2-^ measured using ion exchange (861 Advanced Compact ion chromatograph; Metrohm©) with chemical suppression (ISO 10304-1).

SiO_2_ measured using a Smartchem 200 Discrete Analyzer (WESTCO Scientific Instruments©) (NF T90-007).

Pigments measured in integrated samples using spectrophotometry after extraction into 90% acetone (ISO 10260: 1992).

Specific absorbance measured in the range 200-600 nm in integrated samples filtered on 0.7 µm glass fiber filters and analysed using spectrophotometry.

**Data forms or acquisition methods**: *In situ* profiles and discrete samples for data analyses were collected twice a week during the experiment. Data from the automated probes are provided in the form of csv or txt files and were downloaded after each sampling event. Water samples for chemical analyses were stored at 4°C and the analyses were performed within 48 h after collection.

**Data entry verification procedures**: *In-situ* data are collected and visually checked by the operator after each time of conducting the probes. They are crossed checked by a second operator using the original field data sheets. Laboratory analysis data are collected and manually checked by the operator and validated by authorized scientific staff.

**Quality assurance/quality control procedures**: *In-situ* data are manually validated and crossed validated with laboratory analysis (pH, Cond, dissolved oxygen).

The analytical quality control of laboratory data follows a rigorous traceable workflow from sample collection to data validation. Data are controlled by an analytical quality monitoring of instruments, a verification with reference materials and manual cross-validation between chemist staff.

**Data anomalies**: Negative values of light spectral measurement are detected near 300 nm wavelengths. According to manufacturer’ specifications, negative values can be included in uncertainty interval.

**Computer programs and data-processing algorithms**: For data formatting, homogenization and first visualization we used the software R. Data outliers were mostly manually identified based on quality assurance and quality control procedures (previous section).

## CRediT Author Statement

**Viet Tran-Khac:** Conceptualization, Funding acquisition, Methodology, Software, Data acquisition, Curation, validation, Lab analysis supervision, Writing, Reviewing and Editing; **Philippe Quetin:** Technical support, Methodology, Data acquisition and curation; **Laurent Espinat:** Technical support; **Laura Crepin:** Lab analysis, Data acquisition and curation; **Charlotte Cousin:** PARLAC experiment Lab analysis, Data acquisition and curation; **Pascal Perney:** Technical support, Data acquisition and curation; **Jean-Christophe Hustache:** Technical support; **Geneviève Chiapusio:** DOMLAC experiment Conceptualization, Funding acquisition, Reviewing; **Isabelle Domaizon:** Conceptualisation, Funding acquisition, Supervision; **Serena Rasconi:** Conceptualisation, Funding acquisition, Supervision, Data acquisition and curation, Writing, Reviewing and Editing.

## Declaration of Competing Interest

The authors declare that they have no known competing financial interests or personal relationships, which have, or could be perceived to have, influenced the work reported in this article.

## Data Availability

Physico-chemical and high frequency monitoring dataset from a mesocosm experiment simulating turbid storms by light reduction (Original data) (Dataverse). DOMLAC-Dataset (Original data) (Dataverse).
